# The Relations between the Teeth and the Womb

**Published:** 1885-04

**Authors:** 


### The Relations Between the Teeth and the Womb.

Dr. S. W. Caldwell, of Trenton, Tenn., publishes the following peculiar case in the *Mississippi Valley Medical Monthly*, January 10, 1885:

Recently, while spending the night in the sick-room, I had as company a married daughter of my patient, an intelligent lady, aged about thirty-two years, who related the following--to me—most singular phenomena. Said she: "I am the mother of five living children, one dead. My terms of gestation are unmarked by anything unusual. My labors last about eight hours on an average. My last came as usual with me--pain beginning in the back, passing down to the lower part of my bowels. When I had been in labor about an hour, the pain suddenly ceased in my back and womb, but set up in a tooth that had been aching several times during the past nine months. The pain was paroxysmal, coming and going just as it did in the womb. The pain was in my tooth when my physician came, and he wanted to pull it, but it being one of but few remaining molars, I said to him that the cavity in it was small, and that I wanted to have it filled—couldn't he put something in it and stop the aching? He put cotton saturated with chloroform in the cavity. The pain left the tooth to appear in the back and womb; but the effect soon passed off; the pain in a few minutes returned to the tooth, to be again and again relieved by the chloroform; the doctor said no progress was being made in the labor, and that in his opinion none would be till the tooth was removed."

"Did he say he had ever seen or heard of such a case before?"

"No, sir. Twelve hours had now passed; my former labors had none gone over eight—generally six hours terminated them. I had become, I thought, exhausted, and asked the doctor to pull my tooth, which he did, and my baby was born in an hour."

The attending physician in the above case, and with whom I was personally well acquainted, died not long after, so that I cannot have his history of it. My knowledge of the lady, though, justifies me in giving full credence to her statement.

